# Supplementary material for: Mitochondrial phosphoproteomes are functionally specialized across tissues
Source: Life Sci Alliance. 2023 Nov 20;7(2):e202302147. doi: 10.26508/lsa.202302147 (PMC10662294; doi:10.26508/lsa.202302147)
Supplement: Supplementary file 1 [file LSA-2023-02147_TableS1.docx]

Supplementary Table 1 Overview of mitochondrial phosphoproteome studies in mammalian tissue
The table was adapted and extended from Kruse at al. (Kruse and Hojlund, 2017).

| **Strategy** | **Species** | **Tissue** | **Phosphoproteins and phosphorylation sites identified** | **Reference** |
| --- | --- | --- | --- | --- |
| 2-DE-ProQ staining-MS | Bovine | Heart | 13 phosphoproteins | (Schulenberg et al., 2003) |
| 2-DE-ProQ staining-MS or P32 labelling-2-DE-MS | Pig | Heart | 45 phosphoproteins | (Hopper et al., 2006) |
| IMAC-MS/MS/MS | Mouse | Liver | 84 phosphorylation sites in 62 distinct proteins | (Lee et al., 2007) |
| 2D-BN-PAGE-phos—anti-Ser/Thr/Tyr Ab-MS SCX/IMAC-LC-MS/MS | Rat | Heart | 2-DE: 45 phosphoproteins LC-MS/MS: 26 phosphorylation sites in 19 distinct proteins | (Feng et al., 2008) |
| Anti-Tyr Ab-MS | Rat | Brain | 7 tyrosine-phosphorylation sites in 7 distinct proteins | (Lewandrowski et al., 2008) |
| 32-P labelling/phos-tag 540 gel staining-2-DE-MS | Pig | Liver, Heart | 68 phosphoproteins | (Aponte et al., 2009) |
| iTRAQ labelling-SCX/HILIC/TiO2-LC-MS/MS-HCD | Pig | Heart | 56 phosphorylation sites in 38 distinct proteins | (Boja et al., 2009) |
| IMAC/TiO2-2D-nano LC-MS/MS | Rat | INS-1β cells | 84 distinct phosphoproteins | (Cui et al., 2010) |
| SAX/SCX-LC-MS/MS | Rat | Liver | 447 phosphorylation sites in 228 distinct proteins | (Deng et al., 2010) |
| SDS-PAGE-TiO2-LC-MS/MS | Mouse | Heart | 236 phosphorylation sites in 181 distinct proteins | (Deng et al., 2011) |
| SDS-PAGE-TiO2/CPP/HILIC-LC-MS/MS | Human | Muscle | 155 phosphorylation sites in 77 distinct proteins | (Zhao et al., 2011) |
| IMAC-SCX-nano-LC-MS/MS | Mouse | Liver | 811 phosphosites in 295 distinct proteins | (Grimsrud et al., 2012) |
| TiO2-HILIC-LC-MS/MS | Rat | Liver, Heart, Skeletal muscle | 899 phosphorylation sites in 354 distinct proteins | (Bak et al., 2013) |
| TiO2-HILIC-LC-MS/MS | Human | Skeletal muscle | 207 phosphorylation sites in 95 distinct proteins | (Zhao et al., 2014) |
| iTRAQ labelling-TiO2-LC-MS/MS | Human | Ovary | 124 phosphorylation sites in 67distinct proteins | (Li et al., 2019) |
| Anti-Tyr Ab-MS | Mouse | Liver | 79 tyrosine-phosphorylated mitochondrial proteins | (Guedouari et al., 2020) |
| TMT labeling - IMAC-LC-MS/MS | Human | Skeletal muscle | 480 phosphopeptides that correspond to 196 proteins | (Sathe et al., 2021) |
