# Supplementary material for: Mitochondrial phosphoproteomes are functionally specialized across tissues
Source: Life Sci Alliance. 2023 Nov 20;7(2):e202302147. doi: 10.26508/lsa.202302147 (PMC10662294; doi:10.26508/lsa.202302147)
Supplement: Supplementary file 14 [file LSA-2023-02147_TableS2.docx]

Supplementary Table 2 Phosphorylation on mitochondrial encoded proteins
(nd = protein not detected; x = no phosphorylation detected)

| Gene name | Description | UniprotID | modification |
| --- | --- | --- | --- |
| MT-ATP8 | ATP synthase, Fo subunit 8 (complex V) | P03930 | x |
| MT-ATP6 | ATP synthase, Fo subunit 6 (complex V) | P00848 | x |
| MT-CO1 | Cytochrome c oxidase, subunit 1 (complex IV) | P00397 | x |
| MT-CO2 | Cytochrome c oxidase, subunit 2 (complex IV) | P00405 | x |
| MT-CO3 | Cytochrome c oxidase, subunit 3 (complex IV) | Q7JCX7, P00416 | x |
| MT-CYB | Cytochrome b (complex III) | P00158 | x |
| MT-ND1 | NADH dehydrogenase, subunit 1 (complex I) | P03888 | x |
| MT-ND2 | NADH dehydrogenase, subunit 2 (complex I) | P03893, Q9MD59 | x |
| MT-ND3 | NADH dehydrogenase, subunit 3 (complex I) | P03899, Q7GIP5 | x |
| MT-ND4L | NADH dehydrogenase, subunit 4L (complex I) | P03903, Q7H113 | nd |
| MT-ND4 | NADH dehydrogenase, subunit 4 (complex I) | P03911 | x |
| MT-ND5 | NADH dehydrogenase, subunit 5 (complex I) | P03921 | x |
| MT-ND6 | NADH dehydrogenase, subunit 6 (complex I) | P03925 | *nd |

*Considering that trypsin generally does not cleave N-terminal to proline, tryptic digestion of MT-ND6 does not yield detectable peptides.
